# Identifying pyroptosis- and inflammation-related genes in intracranial aneurysms based on bioinformatics analysis

**DOI:** 10.1186/s40659-023-00464-z

**Published:** 2023-09-27

**Authors:** Donglin Zhou, Yimin Zhu, Peng Jiang, Tongfu Zhang, Jianfeng Zhuang, Tao Li, Linzeng Qi, Yunyan Wang

**Affiliations:** 1 Department of Neurosurgery, Qilu Hospital of Shandong University, Cheeloo College of Medicine and Institute of Brain and Brain-Inspired Science, Shandong University, 107 Wenhua Western Road, Jinan, 250012 Shandong China; 2 Department of Orthopedics, Qilu Hospital of Shandong University, Jinan, China; 3 Jiangsu Center for the Collaboration and Innovation of Cancer Biotherapy, Cancer Institute, Xuzhou Medical University, Xuzhou, China; 4 Department of Neurosurgery, Yangxin County People’s Hospital, Binzhou, China; 5 Department of Neurosurgery, The Third Affiliated Hospital of Shandong First Medical University, Jinan, China

**Keywords:** Intracranial aneurysm, Pyroptosis, Inflammatory cell death, Gene expression omnibus, Bioinformatics analysis

## Abstract

**Background:**

Intracranial aneurysm (IA) is the most common cerebrovascular disease, and subarachnoid hemorrhage caused by its rupture can seriously impede nerve function. Pyroptosis is an inflammatory mode of cell death whose underlying mechanisms involving the occurrence and rupture of IAs remain unclear. In this study, using bioinformatics analysis, we identified the potential pyroptosis-related genes (PRGs) and performed their inflammatory response mechanisms in IAs.

**Methods:**

The mRNA expression matrix of the IA tissue was obtained from the Gene Expression Omnibus database, and 51 PRGs were obtained from previous articles collected from PubMed. The differentially expressed PRGs (DEPRGs) were performed using R software. Subsequently, we performed enrichment analysis, constructed a protein–protein interaction network, performed weighted gene coexpression network analysis (WGCNA) and external validation using another dataset, and identified a correlation between hub genes and immune cell infiltration. Finally, the expression and tissue distribution of these hub genes in IA tissues were detected using Western blotting and immunohistochemical (IHC) staining.

**Results:**

In total, 12 DEPRGs associated with IA were identified in our analysis, which included 11 up-regulated and one down-regulated genes. Gene Ontology and Kyoto Encyclopedia of Genes and Genomes enrichment analyses revealed that the DEPRGs were mostly enriched in the NOD-like receptor signaling pathway, interleukin-1 beta production, and the inflammasome complex. Three hub genes, *NLRP3*, *IL1B* and *IL18*, were identified using Cytoscape software and the WGCNA correlation module, and external validation revealed statistically significant differences between the expression of these hub genes in the ruptured and unruptured aneurysm groups (*p* < 0.05). Furthermore, all AUC values were > 0.75. Immune cell infiltration analysis suggested that the hub genes are related to CD8 T cell, macrophages and mast cells. Finally, IHC staining revealed that the protein levels of these hub genes were higher in ruptured and unruptured IA tissues than in normal tissues (*p* < 0.05).

**Conclusion:**

The results of bioinformatics analysis showed that pyroptosis is closely related to the formation and rupture of IA, and identified three potential hub genes involved in the pyroptosis and infiltration ofcells. Our findings may improve the understanding of the mechanisms underlying pyroptosis in IA.

**Supplementary Information:**

The online version contains supplementary material available at 10.1186/s40659-023-00464-z.

## Background

Intracranial aneurysm (IA), a tumor-like protuberance [1, 2], is characterized by local abnormal vasodilation and vascular wall remodeling. Unruptured aneurysms have a risk of not exhibiting a specific clinical manifestation (unless the aneurysm considerably compresses the cranial nerve). On the other hand, aneurysm rupture triggers subarachnoid hemorrhage that leads to irreversible damage to nerve function [3]. Vascular interventional techniques can effectively prevent and treat IAs; however, the mechanisms of IA formation and rupture remain unclear. Presently, inflammation is considered an important aspect of IA pathophysiology [4]. Change in cerebral hemodynamics induces the chemotaxis of inflammatory cells to the vascular wall [5, 6]. However, whether inflammatory responses are related to the pathological manifestations of IAs, including inner elastic layer rupture, smooth muscle layer loss, and extracellular matrix (ECM) remodeling, remains unclear [2, 7]. Therefore, exploring the mechanism of IA pathogenesis and rupture in terms of molecular biology may provide novel ideas for interventions and rupture delay.

Pyroptosis is a new mode of programmed cell death mediated by the gasdermin (GSDM) protein family [8]. Different from necrosis and apoptosis, pyroptosis involves caspase activation and abundant inflammatory cytokine release, accompanied by a strong inflammatory response and immune cell activation [9, 10]. GSDMs are the main pyroptosis executor. Canonical caspases and granzymes can cleave GSDMs into the gasdermin N and gasdermin C domains after activation by inflammasomes, thereby forming pores in the plasma membrane and causing cell swelling and plasma membrane rupture. This, in turn, promotes the release of inflammatory cytokines such as IL-1β and IL-18 [9, 11]. Furthermore, complex cutting patterns in different GSDM families have been previously revealed, such as gasdermin E (GSDME) cleavage via the apoptotic marker caspase-3 [12, 13]. Pyroptosis plays an active role in immune defense and tumor microenvironment regulation. Vascular smooth muscle cells are critical in IA pathogenesis and help in coping with changes in hemodynamics and maintaining the stability of cerebral blood perfusion. An imbalance in vascular wall homeostasis may lead to changes in the shape of blood vessels, resulting in immune disorders. Developing research has revealed that pyroptosis is associated with vascular dysfunction, including atherosclerosis [14], diabetic microangiopathy, and abdominal aortic aneurysms. Moreover, a study has reported that inhibition of the mouse inflammatory pathway (NLRP3–caspase-1–GSDMD) can effectively reduce abdominal aortic aneurysm occurrence in mice [15]. However, there is a little information concerning pyroptosis in IA.

**Fig. 1:**
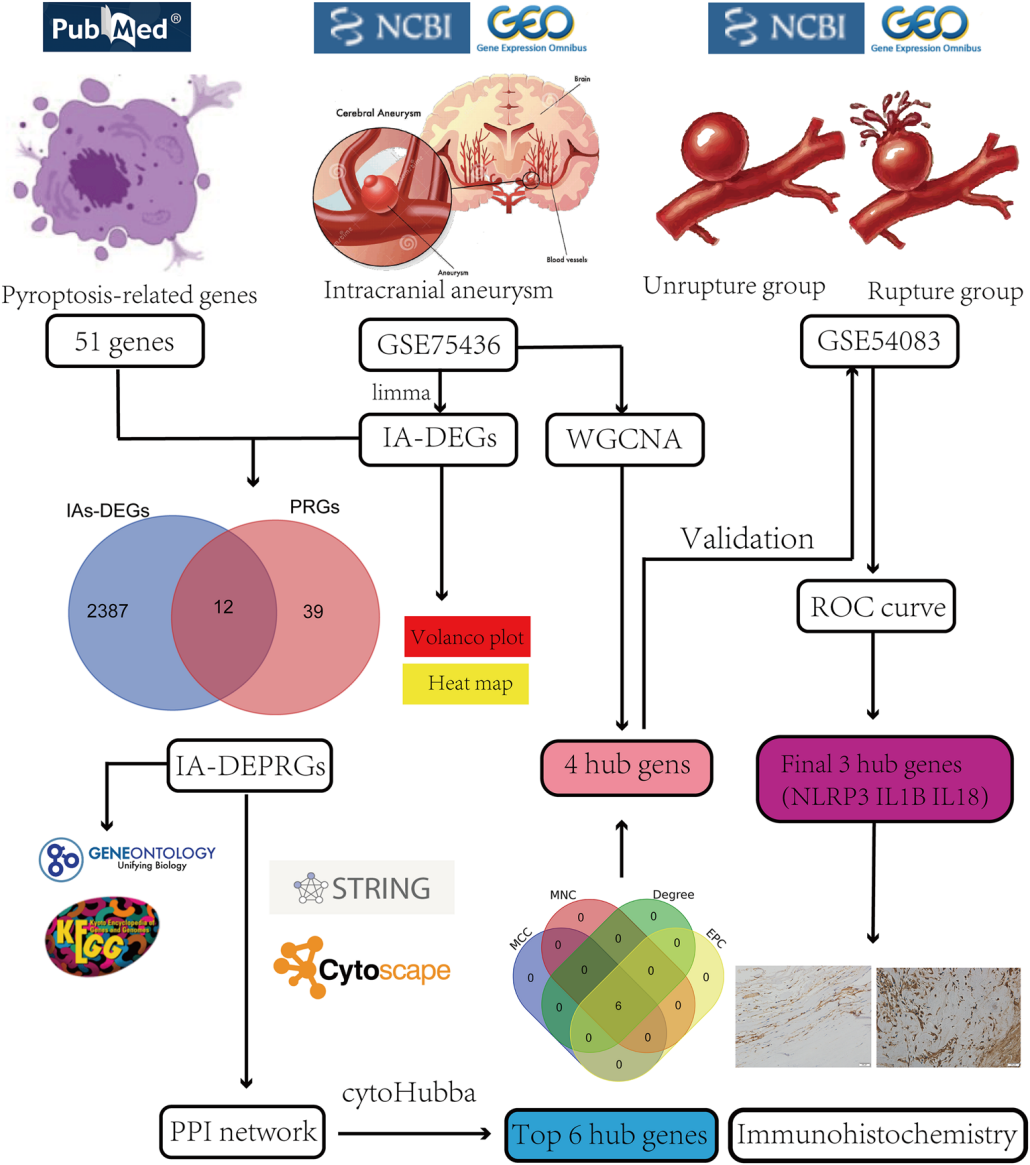
The schematic block diagram of the entire workflow of this study

At present, studies on the mechanisms of PRGs in IA using bioinformatics analysis are lacking. In this study, public datasets were analyzed to screen hub genes and perform immune cell infiltration analysis to explore the mechanism of this novel type of cell death in IA. The purpose was to identify the pathways related to pyroptosis and inflammation in IA, thus providing novel solutions for the diagnosis and delaying aneurysm rupture (Fig. 1).

## Materials and methods

### Data downloading and processing

The terms “intracranial aneurysm” or “IA” and “vessel tissue” and “Homo sapiens” were used to search the National Center for Biotechnology Information Gene Expression Omnibus (GEO) to find the relevant expression data. The GSE75436 [16] and GSE54083 [17] datasets were selected after excluding irrelevant datasets with small sample sizes. The GSE75436 [16] dataset contains 30 samples of unruptured IA tissues and the superficial temporal artery (STA) from 15 patients obtained via aneurysm clipping. The GSE54083 [17] dataset contains 8 ruptured and 5 unruptured IA tissues and 10 STA samples. The gene symbols of the datasets were matched with those of the corresponding GEO platform (GPL). The two datasets that applied the GPL570 and GPL4133 platforms, respectively, were successfully downloaded and proprocessed using the GEOquery R package [18] (version 2.62.2). The deduplication method, which preserves the maximum intensity, was selected when multiple probe sets were annotated by the same gene. The version of R used in our research was 4.1.3. PRGs were collected from previous studies, providing in Additional file 1: Table S1 [19].

### Identifying differentially expressed PRGs (DEPRGs) in IAs

The limma R package (version 3.50.3) was used to perform differential expression analysis of the GSE75436 [16] dataset between the IA and STA groups. The threshold *p*-value was determined by controlling the false discovery rate (FDR), and the corrected *p*-value was the adjusted *p*-value (*Q* value) [20]. The screening criteria were log_2_ |Fold Change|> 1 and the adjusted *p*-value was <0.05. Then, the results were intersected with PRGs to obtain the DEPRGs. The pheatmap package (version 1.0.12) was used to construct the heat map of these genes.

### Enrichment analysis

The distribution trends of the genes and related pathways and molecular mechanisms based on gene expression profiles and phenotypic groupings were evaluated using gene set enrichment analysis (GSEA) [20, 21]. GSEA software (version 3.0) and the Kyoto Encyclopedia of Genes and Genomes (KEGG) gene set (c2.cp.kegg.v7.4.symbols.gmt) were obtained from the Molecular Signatures Database (https://doi.org/10.1093/bioinformatics/btr260, http://www.gsea-msigdb.org/gsea/downloads.jsp) for functional enrichment analysis. The minimum and the maximum gene sets were adjusted to 5 and 5000, respectively. The pathway set list was sorted using the normalized enrichment score (NES). The screening criteria for the significant gene sets were defined as |NES|> 1, *p*-value < 0.05, and FDR *Q*-value < 0.25. Subsequently, Gene Ontology (GO) and KEGG enrichment analyses of the DEPRGs were performed using the ClusterProfiler R package (version 4.2.2). Chord plots and bubble plots were visualized using the SangerBox portal (http://sangerbox.com/). The conversion from gene symbol to Entrez ID before enrichment analysis was performed using the org.Hs.eg.db R package (version 3.14.0).

### Protein–protein interaction (PPI) network construction and hub gene extraction

The DEPRGs were uploaded to the STRINE online database (version 11.5, https://string-db.org/) to predict the interaction relationships between proteins. Then, we downloaded the gene interactive information and embellished PPI network with in the Cytoscape software (version 3.9.1) for better visualization. The molecular complex detection (MCODE) was applied to identify representative interaction subnetwork. The top 6 hub genes were calculated using the four algorithms in the cytoHubba plugin, including degree, Edge Percolated component (EPC), maximum neighborhood component (MNC), and maximal clique centrality (MCC).

### Weighted gene coexpression network analysis (WGCNA)

WGCNA was used to construct a scale-free network by correlating gene expression with clinical features to select the modules with the highest correlation and further screen for hub genes. All genes in the GSE75436 [16] dataset were used for WGCNA in the SangerBox portal after removing deletions and duplicate values. Next, the adjacency matrix was constructed by selecting the appropriate β values under the premise that *R*^2^ is > 0.8 and then transformed into a topological overlap matrix [22]. The dynamic tree cut algorithm was used to identify the module by hierarchically clustering the genes. Subsequently, the correlation between clinical characteristics and modules was assessed. Further screening was achieved by intersecting the genes of the module with the highest gene significance value with pyroptosis-related hub genes.

### External validation

A second dataset, GSE54083 [17], was downloaded from the GEO database for validation studies. It contains unruptured, ruptured, and normal blood vessel samples to verify the differential expression of the hub genes among the three groups. The clinical diagnostic significance of these genes was assessed by plotting the receiver operating characteristic (ROC) curve of the hub genes and calculating the area under the curve (AUC). The final hub genes were selected as the genes with AUCs of > 0.75 and statistically significant expression in the unruptured and ruptured IA groups.

### Analyzing immune cell infiltration and its correlation with pyroptosis-related hub genes

Immune cell infiltration analysis was performed using the CIBERSORT.R script downloaded from the CIBERSORT website [23]. The immueScore results were imported into the SangerBox portal to better visualize the proportion and differential expression profiles of 22 infiltrating immune cell subtypes in IA tissues. The correlation coefficients between each immune cell subtype and between hub genes and infiltrating immune cells were calculated, and the results were then visualized using correlation heatmaps and line graphs, respectively.

### Tissue specimens

The tissues of 8 IAs (4 unruptured and 4 ruptured) and 4 STAs from patients who underwent aneurysm clipping and STA–middle cerebral artery bypass at Shandong University Qilu Hospital, China, were collected. Detailed clinicopathological information is provided in Additional file 2: Table S2. The study was strictly conducted according to the principles of the Declaration of Helsinki and approved by the Ethics Committee of Shandong University Qilu Hospital. Informed written consent was obtained from the patients.

### Protein extraction and Western blotting

Western blotting was performed to validate the differential protein levels of the relevant hub genes in unruptured IAs and normal vessels. Clinical tissue samples were sufficiently cleaved using the total protein extract solution and a homogenizer to extract proteins according to the manufacturer’s instructions (Bestbio, Nanjing, China). After measuring the protein concentrations using a bicinchoninic acid protein assay kit (Bytotime, Shanghai, China), equal amounts of protein were separated using 12.5% sodium dodecyl sulfate–polyacrylamide gel electrophoresis (EpiZyme Shanghai, China) and transferred onto a polyvinylidene fluoride membrane (Merck Millipore, Darmstadt, Germany). The following antibodies were used for Western blotting: rabbit anti-human NLRP3 (HUABIO; #SC06-23), rabbit anti-human IL-1β (Proteintech; #16806-1-AP), and rabbit anti-human IL-18 (Proteintech; #10663-1-AP) antibodies. The membranes were then developed and visualized using the SuperKine^TM^ECL substrate (Abbkine, Wuhan, China) and the ChemiDocTM MP Imaging System (Bio-Rad, California, USA), respectively. The band intensity of the proteins was quantified by using ImageJ, and the relative protein level was obtained.

### Immunohistochemical (IHC) staining

IHC staining was performed to determine the expression of key genes and their cellular localization. Briefly, paraffin sections were incubated with NLRP3, IL-1β, and IL-18 antibodies (antibodies same as that used for Western blotting) overnight at 4 °C after dewaxing and antigen repair. Then, the sections were incubated with rabbit anti-goat secondary antibodies labeled with corresponding horse radish peroxidase (Cell Signaling Technology, Massachusetts, USA) at 25 ℃ for 30 min. Next, the sections were stained with 3,3′-diaminobenzidine and counterstained with hematoxylin (Gene Tech, Shanghai, China) and then visualized. The mean optical density (MOD) values were calculated using Image Pro Plus (version 6.0).

### Statistical analysis

Using the online databases and R package mentioned above, statistical analysis was partially automated. GraphPad Prism9 software was used to obtain data statistics and charting of IHC staining and Western blotting. Differences between two groups and multiple groups were calculated using the *t*-test and one-way analysis of variance, respectively. Non-parametric tests are used when the data does not conform to normal distribution. The results were expressed as mean ± standard error of mean. Correlation analyses were performed using Spearman correlation analysis. A *p*-value of < 0.05 was considered statistically significant.

## Results

### Identifying DEPRGs

**Fig. 2:**
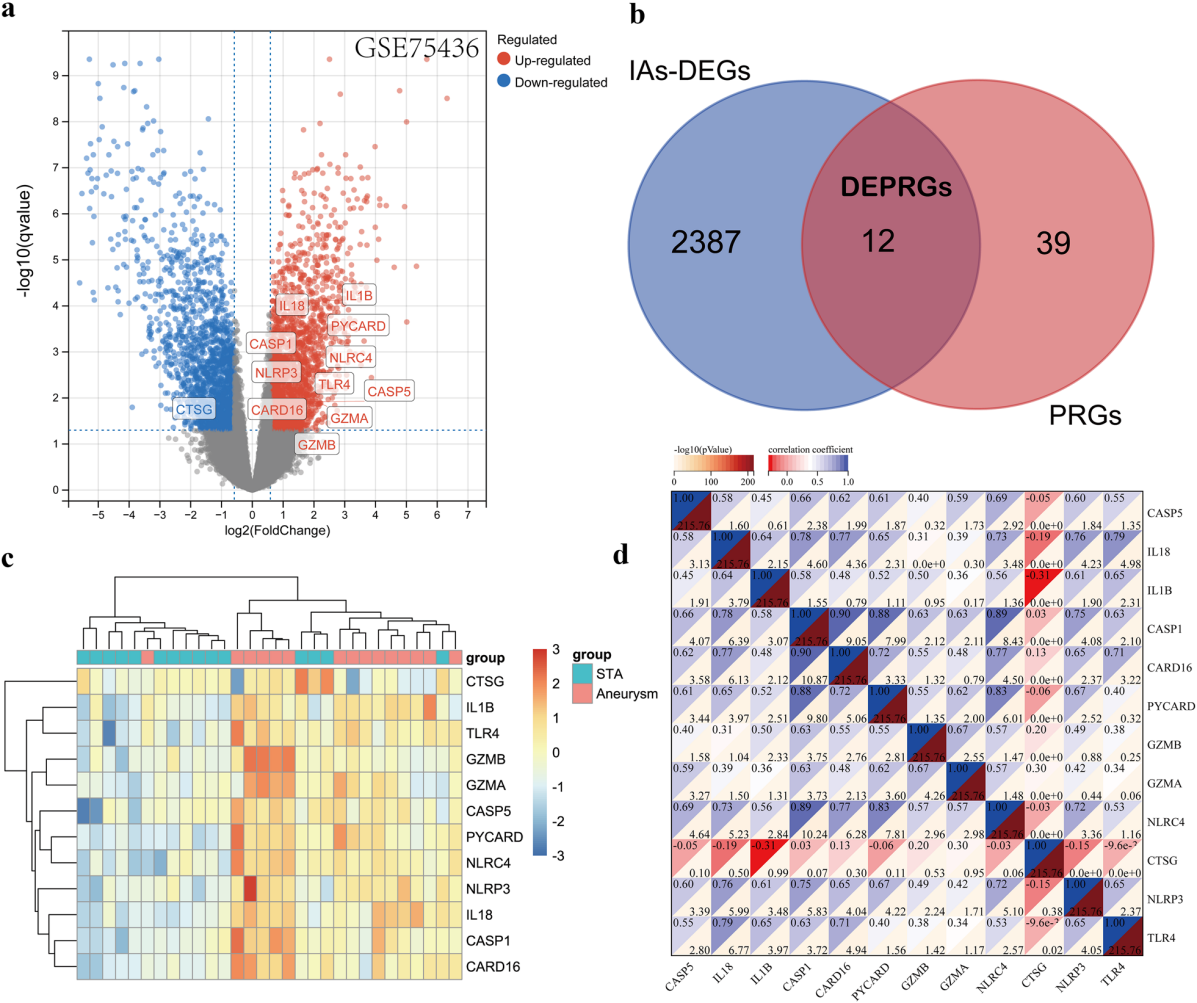
Identification of DEGs and DEPRGs in IAs. **a** The volcano plot of the 2,899 DEGs in the GSE75436. The *x* axis is log_2_|FC| while the y axis is -log(adjusted *P*-value).The red dots represent upregulated genes and the blue dots represent downregulated genes, whereas gray dots represent nonsignificant expressed genes. Adjusted *P*-value < 0.05 and |log2 fold change|> 1. **b** Identification of 12 DEPRGs from PRGs and DEGs. **c** The heatmep of 12 DEPRGs in GSE75436 [16]. Each row represents a gene and each column represent a sample. **d** Correlation heatmep of 12 DEPRGs. There was a strong correlation among the 12 DEPRGs

Compared with genes in the STA sample, 2399 genes with significant changes in the IA patients tissues from the GSE75436 [16] dataset were identified by screening criteria, as shown in the volcano plot (Fig. 2a) and organized in Additional file 3: Table S3. In total, 51 PRGs were collected from published articles in the database [20], including genes regulating the expression of PRGs and inflammasomes. The 12 DEPRGs extracted from intersection of the PRGs with these DEGs using venn diagram (Fig. 2b), which consist of one down-regulated gene and 11 up-regulated genes. The expression of these genes was plotted as heat map (Fig. 2c). Then, the correlation analysis confirmed a good correlation among these DEPRGs (Fig. 2d). IL1B and CTSG exhibited a distinct negative correlation (*r* =  − 0.31, *p* < 0.05), whereas CASP1 and CARD16 were highly positively correlated (*r* = 0.90, *p* < 0.05).

### Enrichment analysis

**Fig. 3:**
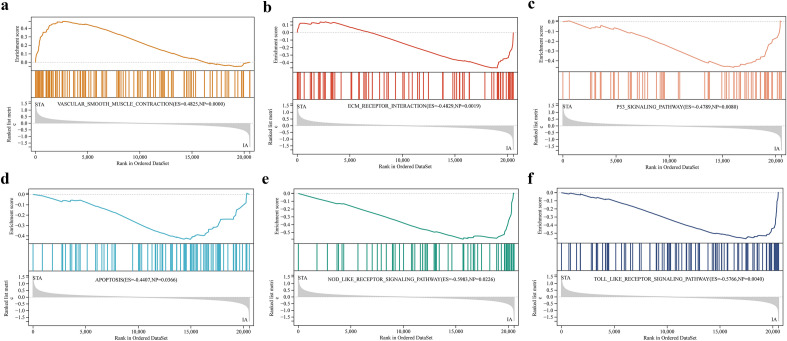
GSEA plots showing most enriched biological pathway in in the IAs group compared with the STAs group.** a** The first significant enriched gene set is vascular smooth muscle contraction (ES = 0.483, NES = 1.654, *P* < 0.05). **b** The second significant enriched gene set is ECM receptor interaction (ES = − 0.483, NES = − 1.601, *P* < 0.05). **c** The third significant enriched gene set is p53 signaling pathway (ES = − 0.479, NES = − 1.689, *P* < 0.05). **d** The fourth significant enriched gene set is NOD like receptor signaling pathway (ES = − 0.598, NES = − 1.624, *P* < 0.05). **e** The fifth significant enriched gene set is apoptosis (ES = − 0.441, NES = − 1.5715, *P* < 0.05). **f** The sixth significant enriched gene set is Toll like receptor signaling pathway (ES = − 0.576, NES = − 1.709, *P* < 0.05)

**Fig. 4:**
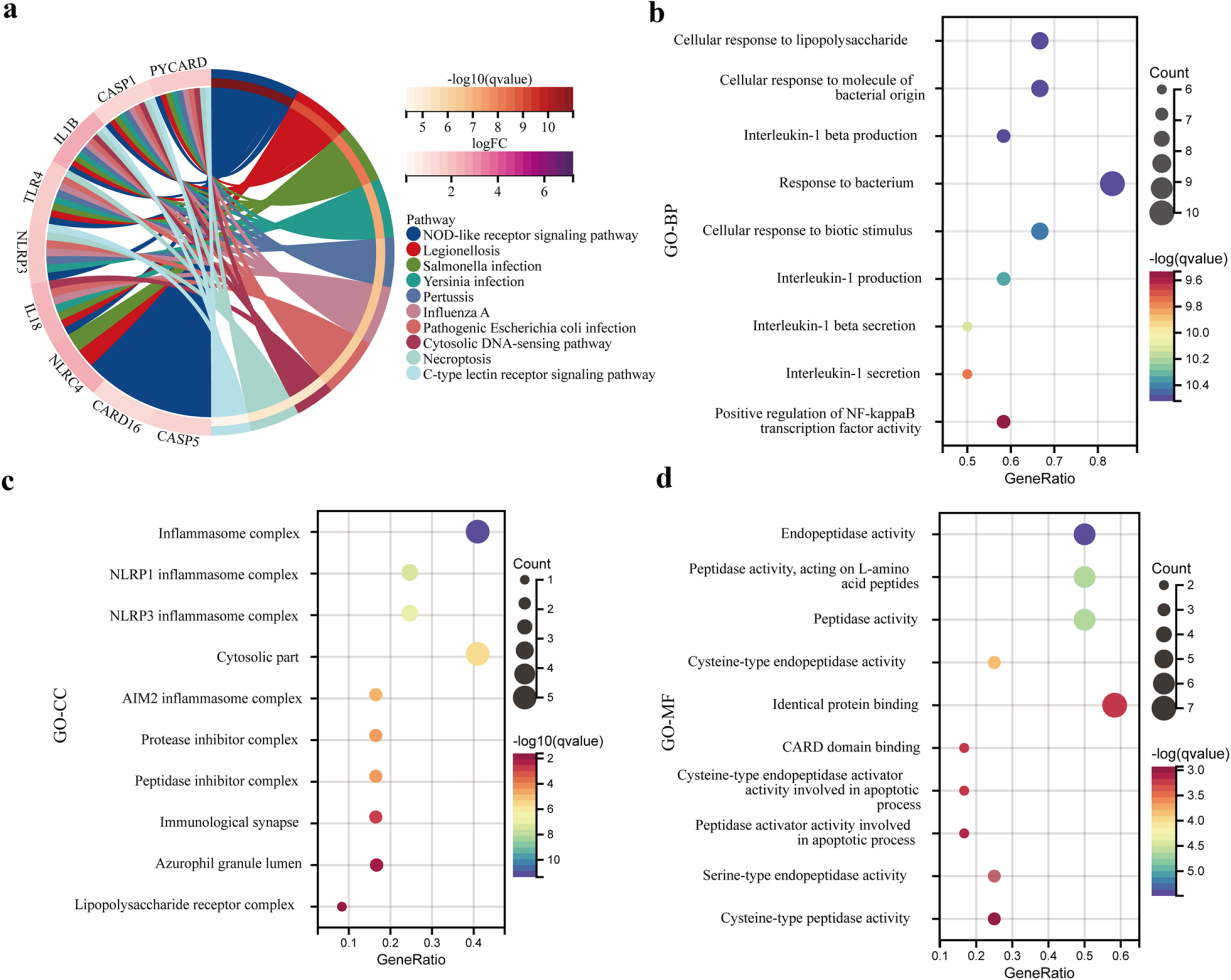
KEGG and GO enrichment analysis of 12 DEPRGs. **a** The chord plot showing the top10 enriched KEGG pathway of DEPRGs. NOD-like receptor signaling pathway is most notable. **b–d** GO enrichment analysis, **b** biological process (BP), c cellular components (CC), **d** molecular function (MF). Bubble plots showing the top 10 information involved in each GO term. These indicate that compared with STA, IA tissue has stronger activation of pytoptosis pathway members, such as inflammasome complex and cysteine-type endopeptidase. The *q* value is the adjusted *P* value

The potential biological pathways involved in gene expression in IAs were explored using the GSEA method. The result reveal that most of the enriched gene sets were mainly related to the vascular smooth muscle contraction, process of cell proliferation and apoptosis, and Immune inflammatory response pathway (Fig. 3). GO and KEGG pathway analyses were subsequently performed to enrich the DEPRGs to further understand their molecular functions and signaling pathways from multiple perspectives. The most significant enrichment terms analyzed using KEGG were NOD-like receptor signaling and C-type lectin receptor signaling pathways, and these pathways were also indicated in the conclusions of GSEA.We selected the top 10 KEGG pathways visualized as a chord plot (Fig. 4a). Furthermore, the results of GO enrichment analysis revealed that the pyroptosis process and inflammatory response may be involved in the occurrence of IAs, and its specific pathways may mainly include interleukin-1 beta production (biological process), NLRP–inflammasome complex (cellular component), and cysteine-type endopeptidase activator activity, as well as in apoptosis (molecular function). In particular, biological processes such as the positive regulation of NF-κB transcription factor activity and cellular response to the molecule of bacterial origin were significantly enriched (Figs. 4b–d). The most significant pathways obtained by GSEA, GO and KEGG analysis were shown in Additional file 4: Table S4 and Additional file 5: Table S5.

### PPI network construction

**Fig. 5:**
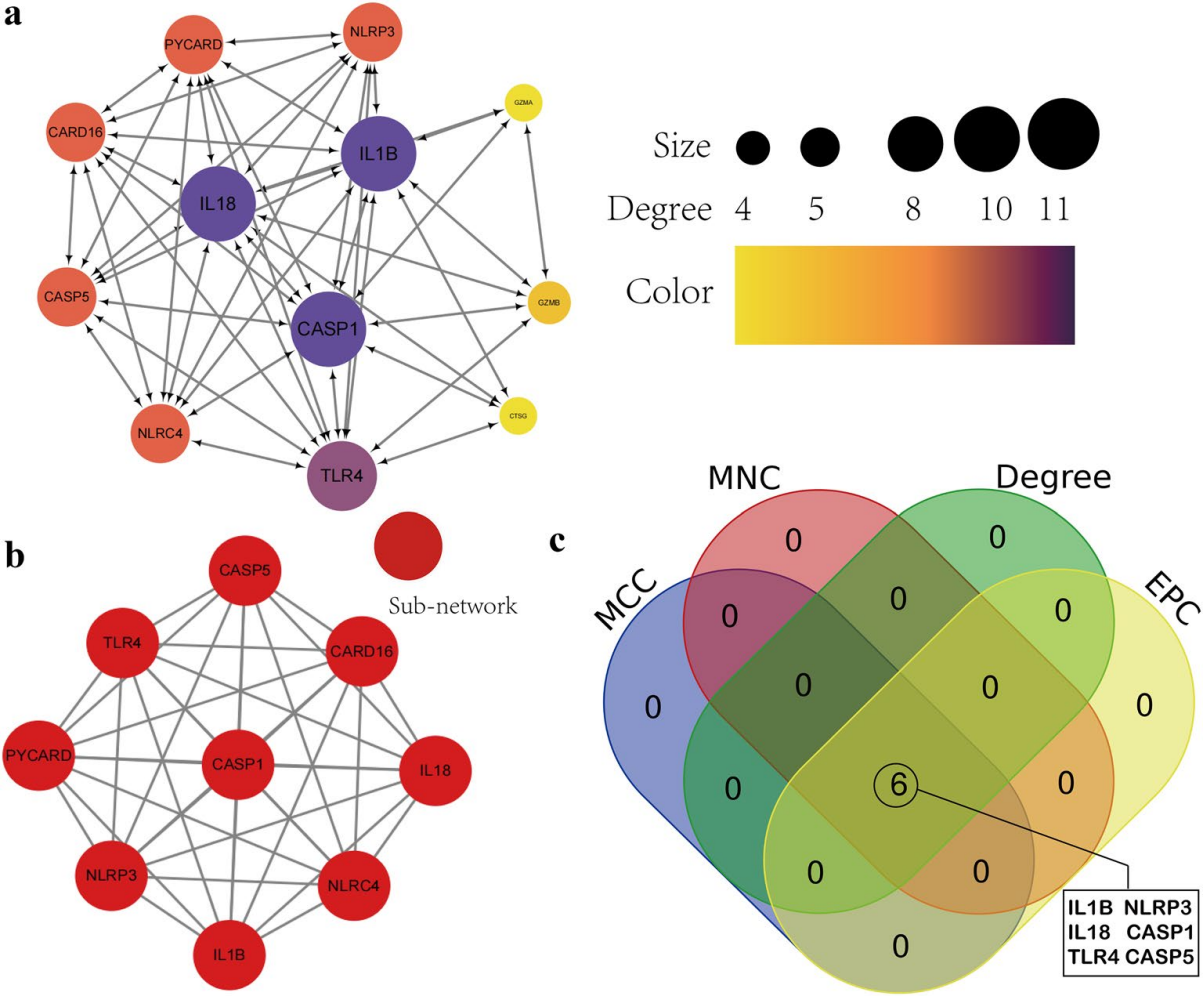
Construction of PPI network of DEPRGs and identification of hub genes **a** Using the STRING online database, the PPI network of 12 DEPRGs was constructed, concluding 12 nodes and 48 edges. Each node represents a protein and each edge represents the interaction between two proteins. **b** Identification of only one sub-network by MCODE plugin in Cytoscape. **c** Identification of 6 hub genes with the highest degree identified by four algorithms (MCC, MNC, Degree and EPC) from CytoHubba plugin in Cytoscape

Next, we constructed a network graph using the STRING online tool to investigate the potential relationship of proteins encoded by the DEPRGs. The network contained 12 nodes and 48 edges, and the average node degree was 8, which nodes denote genes, edges denote gene interactions (Fig. 5a). This implies a close interaction between these DEPRGs. We also acquired only one sub-network with high clustering scores (Fig. 5b). Then, the top six hub genes (*CASP1, NLRP3, CASP5, TLR4, IL1B*, and *IL18*) were picked out from 12 nodes using a combination of four algorithms (degree, EPC, MCC, and MNC) in the cytoHubba plugin of Cytoscape software (Fig. 5c). The topological analysis and node and edge information of the PPI network are supplemented in Additional file 6: Tables S6–S8.

### WGCNA method

**Fig. 6:**
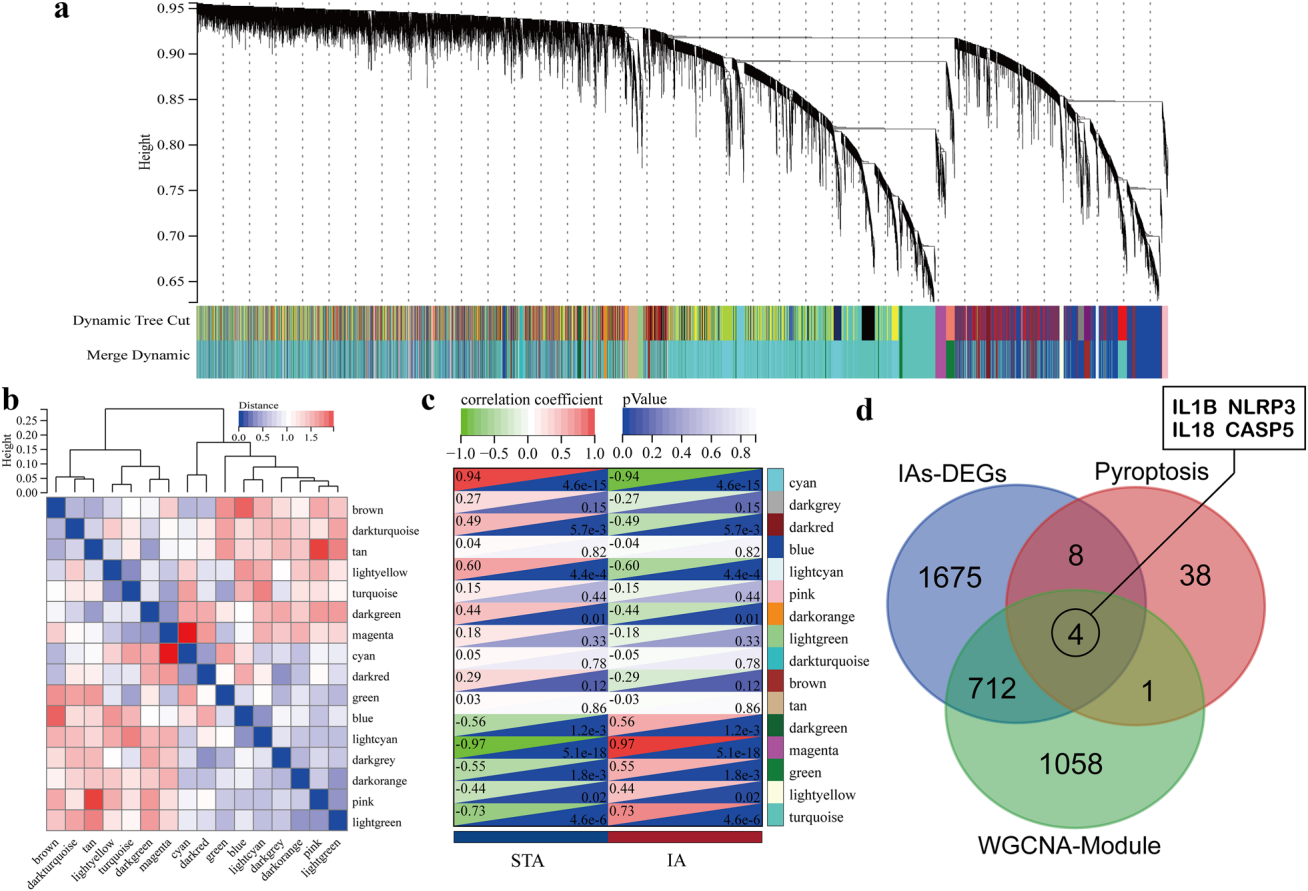
Weighted correlation network analysis. **a** Recognition module, each module was given an individual color as identifiers, including 15 different modules. **b** Clustering of module eigengenes and correlation between different modules. **c** Correlation heat map of gene modules and phenotypes. This demonstrates that cyan is negatively correlated with the phenotype; magenta is positively correlated with the phenotype in IA group. **d** The genes associated with clinical phenotypes was obtained by taking the intersection of DEG, pyroptosis gene and two module genes, showing as Venn diagram

The WGCNA method was used to identify the key modules to further screen the hub genes. We chose 2 as the *β* value as that corresponds to *R*^2^ value of 0.87 to construct scale-free network (Additional file 7: Figure S1a, b). Using the dynamic tree cut algorithm, a total of 11 coexpression modules were identified and the number of genes in these modules ranged from 53 to 4005 (Fig. 6a). The connectivity was calculated and cluster analysis was performed among the 11 modules (Fig. 6b). Further, to verify the relationship between the modules and clinical phenotypes, we calculated the correlation coefficients to reflect each module and IA characteristics. The results showed that the magenta (*r* = 0.97, *p* < 0.001) and cyan (*r* = − 0.94, *p* < 0.001) modules had the strongest positive and negative correlation with IAs, with 142 and 1633 genes, respectively (Fig. 6c). The scatter plots of two eigengene modules were provided in Additional file 7: Figure 1c, d. Subsequently, we intersected these two modules with PRGs and DEGs to obtain the PRGs related to clinical phenotypes. We observed that four genes *NLRP3*, *IL18*, *IL1B*, and *CASP5*, overlapped in a venn diagram (Fig. 6d). Coincidentally, all these four genes were included in the top 6 hub genes selected form PPI network. Therefore, follow-up studies were based on these four genes as hub genes associated with clinical phenotypes.

### External validation of the hub genes

**Fig. 7:**
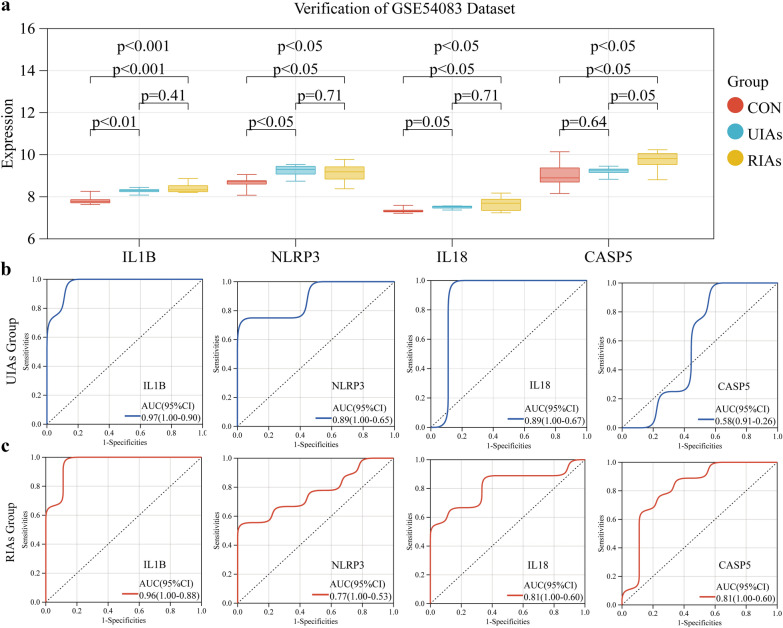
External validation of hub genes. **a** The expression of 4 hub genes (IL1B, NLRP3, IL18 and CASP5) in different states of aneurysms, including unruptured and ruptured, in the GSE54083 dataset. **b** ROC curve of the 4 hub genes in unruptured aneurysms (UIAs) group. **c** ROC curve of the 4 hub genes in ruptured aneurysms (RIAs) group. AUC area under the ROC curve

We examined the expression and accuracy of the hub genes in different states of the IAs in another IA dataset GSE54083 [17]. The IA samples in this dataset were divided into two groups, namely, the unruptured (UIAs) and ruptured (RIAs) groups, and compared. Consistent with the findings of differential analysis in the GSE75436 [16] dataset, we indicated that except for *CASP5*, *NLRP3, IL1B*, and *IL18* were significantly upregulated in the UIAs group (*p* < 0.05). In contrast, the expression of *CASP5* was different in the RIAs group than in normal blood vessels (Fig. 7a). Nevertheless, the expression of other hub genes in the RIAs group was not significantly different from that of those in the UIAs group; however, it was generally higher than that of those in the control group. To explore the effectiveness of these four genes as potential biomarkers for IA, we also performed ROC curve analysis, with the *x*-axis and *y*-axis indicating sensitivity and specificity, respectively. The larger the AUC, the more accurate the diagnostic model. The AUCs for *IL1B*, *NLRP3*, *IL18*, and *CASP5* were 0.97, 0.89, 0.89, and 0.58 in the UIAs group and 0.96, 0.77, 0.81, and 0.81 in the RIAs group, respectively. Taken together, the results suggest *IL18*, *IL1B*, and *NLRP3* as good diagnostic markers for predicating IAs formation and rupture owing to nonsignificant differences in *CASP5* expression (Fig. 7b, c).

### Analysis of immune cell infiltration

**Fig. 8:**
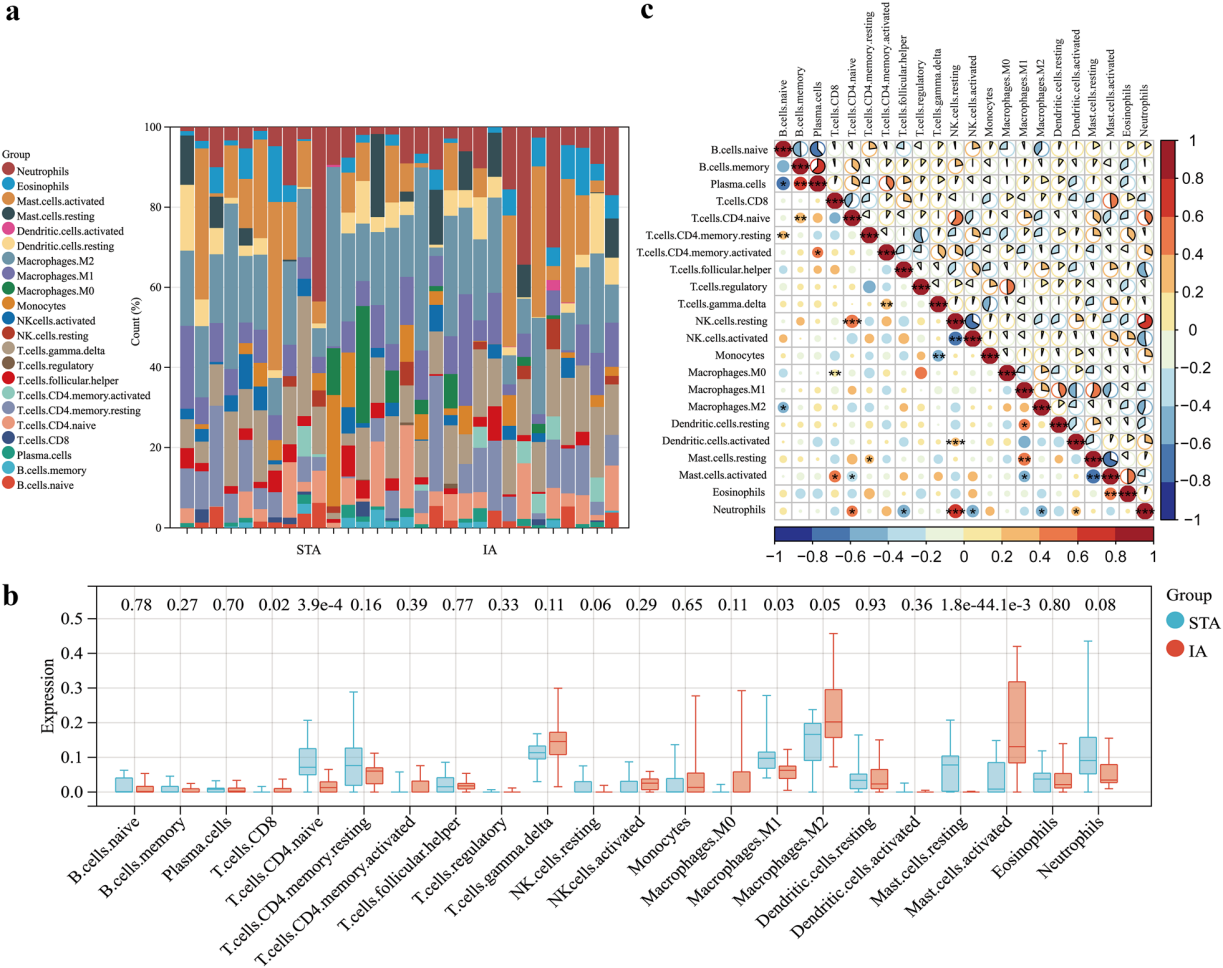
Evaluation and visualization of immune cell infiltration. **a** immune cell types and rations of IA tissues. **b** Boxplot diagram of the 22 types of immune cells between IAs and STAs group. **c** Correlation heatmap of 22 types of immue cell. The size of the pie chart at the top right and the circle at the bottom right represent the Spearman correlation coefficient. The range of *P*-values is indicated by asterisks. The red and blue represent positive and negative correlation (*P < 0.05; **P < 0.01; ***P < 0.001; ****P < 0.0001)

**Fig. 9:**
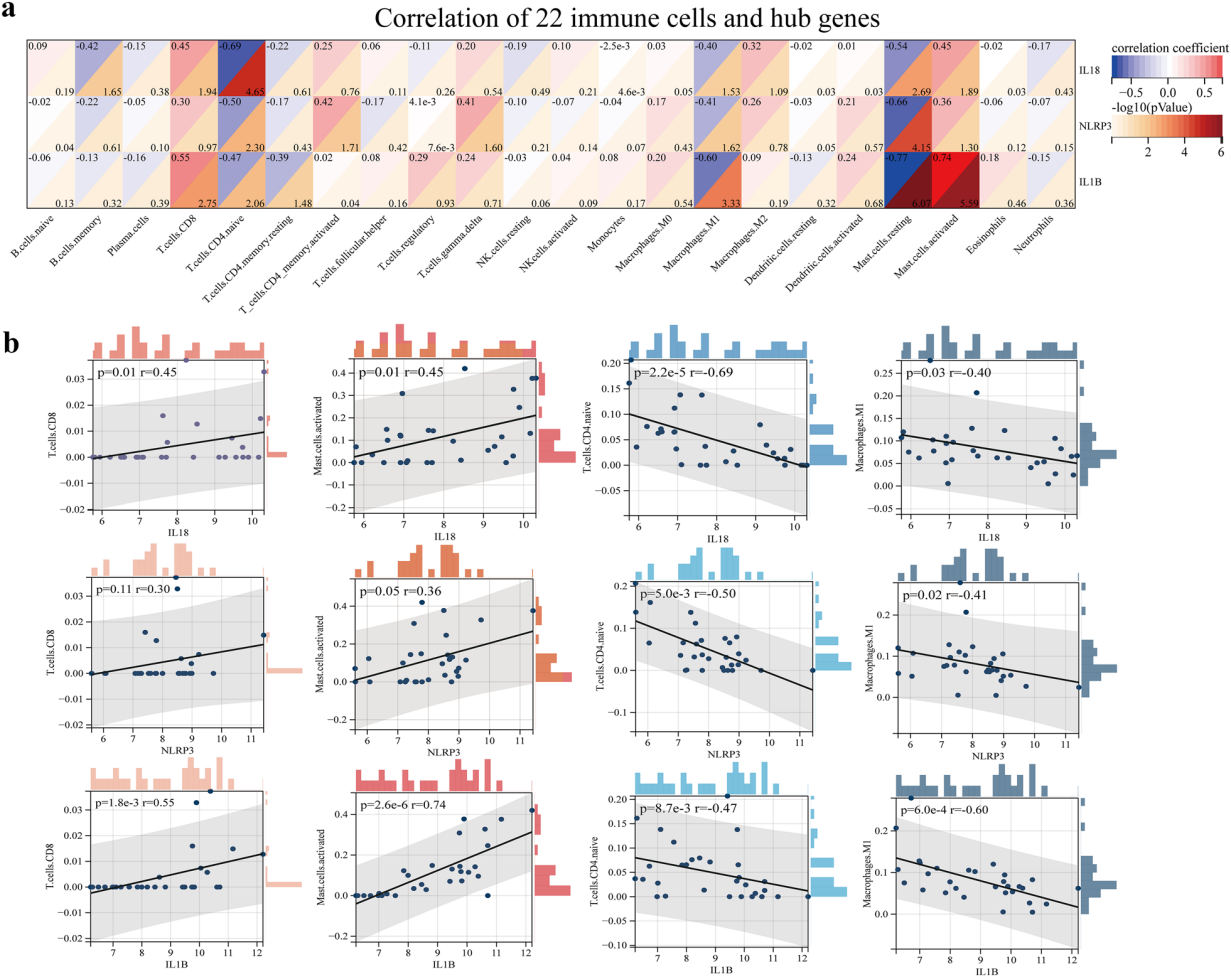
Correlation analysis of final three hub genes and immune cells. **a** Correlation heat map between final hub genes (IL18, NLRP3 and IL1B) and 22 kinds of immune cells. The results showed that these hub genes were mainly associated with CD8 T cells, activated mast cells, naive CD4 T cells and M1-type macrophages. **b** Correlation scatter plots of final hub genes and four immune cells

Pyroptosis is closely associated with immune microenvironment development. To further explore the differential gene expression of the immune fractions between IA tissues and the STA, we used the CIBERSORT algorithm to calculated immune score of each sample in the GSE75436 [16] dataset. The relative proportion of the 22 immune cell subtypes is presented in the cumulative histogram (Fig. 8a). We surprisingly observed a relatively large proportion of mast cells, CD8 T cells, and macrophages, indicating that these immune cells participate in the formation of IAs. We further analyzed the expression of these 22 kinds of immune cells subtypes in the databases and examined their differences. The result reversed that the proportion of resting mast cells was decreased (*p* < 0.001) but that of active mast cells was significantly increased (*p* < 0.01) in IA than in normal vessels, which indicates that it exists transformation of mast cells to an activated situation in IA tissues (Fig. 8b). Then, in immune cell correlation analysis, active mast cells were positively correlated with eosinophils and CD8 T cells that indicates the function of activated mast cells, eosinophils and CD8 T cells in IA may synergistic, but negatively correlated with resting mast cells. Further, the relationship between M2 macrophages and neutrophils may be antagonistic (Fig. 8c). Immunoinfiltration score and correlation analysis data were provided in Additional file 8: Tables S9–S10. Next, we performed correlation analysis between the hub genes and infiltrated immune cells; the results are presented as correlation heat maps in Fig. 9a. IL1B was positively correlated with active mast cells (*r* = 0.74, *p* < 0.001) and CD8 T cells (*r* = 0.55, *p* < 0.001) and negatively correlated with resting mast cells (*r* =  − 0.77, *p* < 0.001). The correlation trend of IL18 and NLRP3 was also partially similar, demonstrating a positive correlation with active mast cells (*r* = 0.45, *p* < 0.01 and *r* = 0.36, *p* < 0.05, respectively) and a negative correlation with resting mast cells (*r* =  − 0.54, *p* < 0.001 and *r* =  − 0.66, *p* < 0.001, respectively) and M1 macrophages (*r* =  − 0.40, *p* < 0.05 and *r* =  − 0.41, *p* < 0.05, respectively; Fig. 9b).

### Validation of the hub genes using Western blotting and IHC staining

**Fig. 10:**
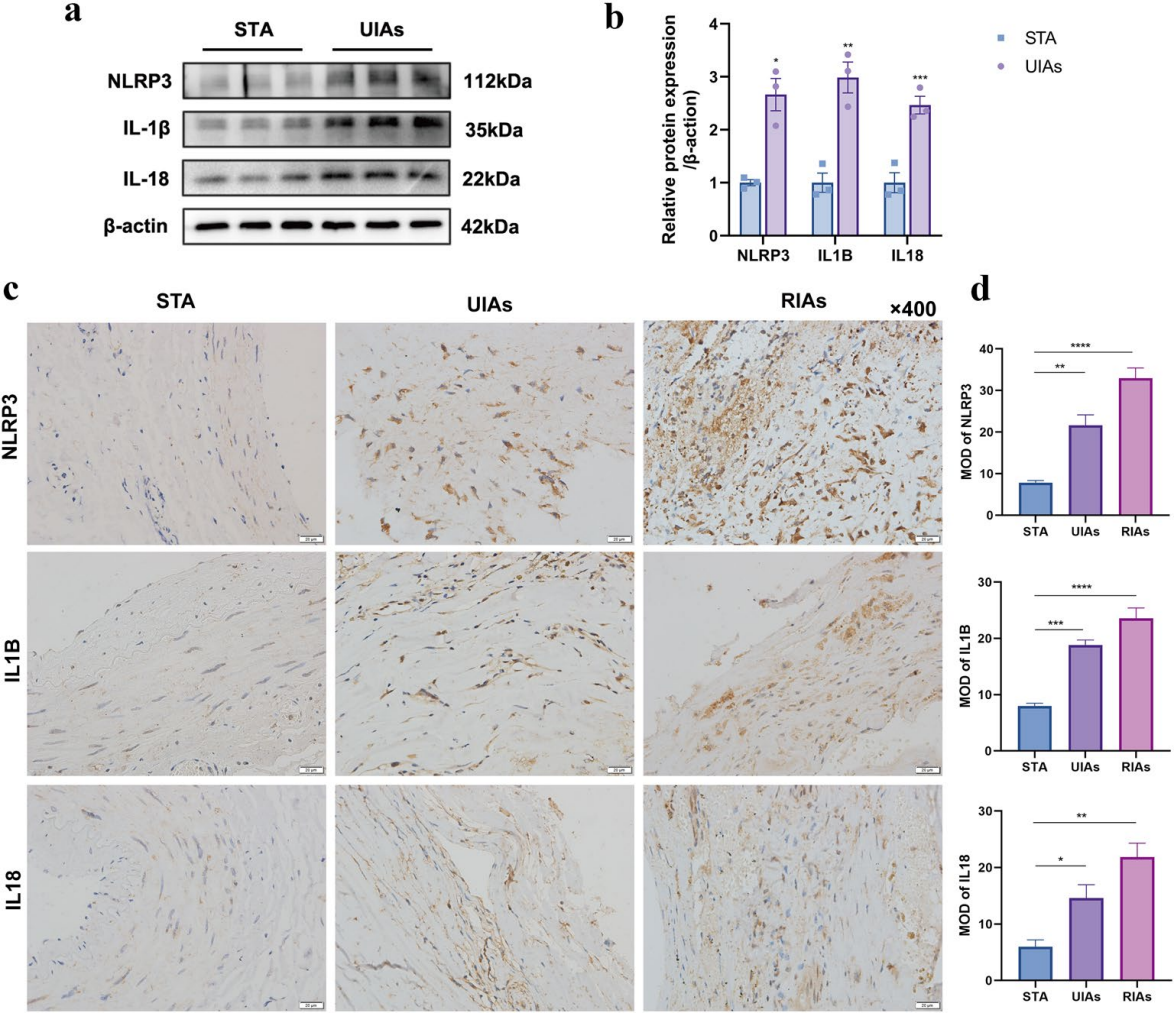
Expression of hub genes in intracranial aneurysm and superficial temporal artery (STA) group. **a, b** Western Blot results indicated that the three hub genes expression in unrupture aneurysms group was higher than that in STA group. **c, d** immunohistochemistry results showed that there was a significant increase in the expression of hub genes in both the unruptured and ruptured groups, all of which were concentrated in the cytoplasm. The positive staining of smooth muscle, endothelial cells and inflammatory cells was evident (*P < 0.05; **P < 0.01; ***P < 0.001; ****P < 0.0001)

The hub genes *IL1B*, *IL18*, and *NLPR3* were mainly localized in the cytoplasm. First, we performed Western blotting of the tissues from patients with unruptured IAs and normal vessels to validate the expression of proteins corresponding to these hub genes. Our result confirmed that the protein levels of IL1B, NLRP3, and IL18 were higher in the UIAs than in normal vascular tissues (*p* < 0.05; Fig. 10a, b) Further, we performed IHC staining of the UIAs and RIAs tissue sections to explore the cellular distribution of their proteins. The arrangement of vascular media was disordered, the number of smooth muscle cells decreased and cell morphology changed in the AI samples. A large numbers of erythrocytes and inflammatory cells were invaded in the UIAs tissues. The MODs of IL1B, IL18, and NLRP3 proteins were all higher in IA than in normal vascular tissues (*p* < 0.05). These proteins may locate in smooth muscle cells, endothelial cells, and inflammatory cells (Fig. 10c, d).

## Discussion

In this research, we analyzed the differential gene expression of IA and normal vascular tissues and obtained the differential expression profiles of PRGs in IA, including 11 upregulated genes and 1 downregulated gene. *NLRP3* is one of the marker genes of the classical pyroptosis pathway, indicating that pyroptosis is related to IA pathogenesis. To clarify the involvement of the identified genes in IA pathogenesis, we performed enrichment analysis. We observed that these genes were primarily involved in biological processes in inflammatory bodies. Further, we analyzed immune cell infiltration in IA tissues to investigate the relationship between key genes and immune cells. We observed that mast cells, T cells, and macrophages are involved in the immune regulation of IA pathogenesis. Moreover, we used external datasets and experimental validation results to confirm the reliability of bioinformatics analysis. Taken together, our study results may provide a new direction for studying IA pathogenesis.

Analysis of differential genes associated with pyroptosis in IA, based on bioinformatics analysis, appears to point to an inflammatory response. IAs are considered to be the result of an imbalance in the environmental homeostasis of the arterial wall, pathologically characterized by smooth muscle layer stabilization and elastic fiber rupture. Many studies have mainly focused on the role of inflammatory responses in the pathogenesis of aneurysms. A Study on intestinal flora of intracranial aneurysm have confirmed that *Hungatella hathewayi* is the only strain that is associated with taurine; it effectively inhibits cerebrovascular inflammation and extracellular matrix remodeling [24]. The change of hemodynamic changes are the initial factors for the formation of IA, which may promote the chemotaxis of inflammatory cells and cause local vascular inflammatory response. [6, 25]. Further, the transformation of smooth muscle cells from the contractile phenotype to the proinflammatory/promatrix remodeling type in IA tissues can be considered as response of vascular inflammation [26]. In addition, elevated levels of inflammatory cytokines and chemokines, such as TNF-α, IL-6 [27, 28], and TGF-β [29], were detected in the blood and cerebrospinal fluid of patients with IA. Taken together, these studies suggest the important role of immune responses in IA formation. And whether these inflammatory processes in IA are connected with pyroptosis requires further exploration.

Pyroptosis is an inflammatory form of programmed cell death that is often accompanied by cell lysis, contributing to the release of proinflammatory cytokines [14]. The pyroptosis of macrophages [30] and smooth muscle cells [31] plays a dominant role in the pathogenesis of isovascular degeneration in atherosclerosis [32] and abdominal aortic aneurysms [33]. However, whether the inflammatory responses in IA pathogenesis are related to pyroptosis remains unclear. Here, we explained the molecular characteristics of pyroptosis in IA in detail by comprehensively analyzing public datasets.

In the present study, genome-wide GSEA analysis revealed that the genes in IA samples were mainly concentrated in smooth muscle systolic dysfunction, ECM receptor interaction, and cell proliferation inhibition and apoptosis. This reveals important information regarding the mechanisms underlying IA formation. Therefore, anything that affects these biological processes may contribute to aneurysm development. We are surprised to find that this results partially coincide with the KEGG and GO enriched pathway. All three enrichment methods identified that NOD-like receptors along with toll-like receptor (TLR) and C-type lectin families constitute the immune recognition system [34], which plays a critical role in inflammation and metabolism. NOD-like receptors such as NLRP1, NLRP3, and NLRC4 participate in the formation of inflammatory bodies that activate the process of pyroptosis [35]. KEGG pathway analysis also revealed that six DEPRGs were associated with a response to infection, with pathogens such as *Salmonella* and *Escherichia coli*. This is consistent with the bacterial profile of infectious IAs reported by Kannoth et al. [36, 37]. In fact, the process of pyroptosis can be activated by extracellular and intracellular stimuli, such as bacteria and viruses [38], which plays an important role during bacterial infection [39]. Some studies have found that *Salmonella* can induce and regulate caspase-1-dependent pyroptosis in macrophages [40], and macrophages exposed to outer membrane vesicles from uropathogenic *E. coli* can activate NLRP3 inflammasome after inducing mitochondrial apoptosis [41]. Infectious aneurysms can rapidly expand [36], and pathological examination has revealed destruction of the inner elastic layer and infiltration of the smooth muscle layer of inflammatory cells [36, 43]. Therefore, pyroptosis induced by bacterial inflammatory responses may be related to the rapid progression of IA, but this inference needs further experimental support. Another GO-enriched biological process that was identified was the positive regulation of NF-κB transcription factor activity, suggesting that inflammatory responses in IAs regulate NLRP3 to induce pyroptosis via the NF-κB pathway [44]. Several studies have reported that high wall shear stress blood flow activates this pathway in the endothelial and smooth muscle cells and promote macrophage chemotaxis [26, 45]. Therefore, we can speculate that the shock of blood flow on the vessel wall may promote the formation of IA by inducing pyrotposis and inflammatory responses in the vascular cell.

We constructed a protein–protein interaction network using the STRING database and identified the top six hub genes that are related to pyroptosis among the DEPRGs in IA using Cytoscape. The top six hub genes were *CASP1, IL1B, IL18, TLR4, CASP5,* and *NLRP3.* Although TLR4 expression was not screened in the WGCNA correlation module, it plays an important role in the regulation of vascular oxidative stress and neuroinflammation. As a member of the TLR family, TLR4 is one of the TLRs expressed on the surface of smooth muscle, endothelial, and immune cells. TLR4 binds to myeloid differentiation primary-response protein 88 (MyD88), thereby activating IL-1R-associated kinases and expressing downstream proinflammatory cytokines [46, 47]. Recent studies have shown that inhibition of the TLR4 pathway in inflammatory cells reduced IA rupture [46]. Interestingly, the TLR4/NF-κB pathway plays an essential role in regulating neuronal apoptosis and cerebral vasospasm after aneurysmal subarachnoid hemorrhage [48].

Surprisingly, we found that these hub genes were concentrated in the classical cell inflammatory death pathway, namely the caspase-1-dependent pyroptosis pathway [49]. NLRP3-mediated inflammatory bodies activate caspase-1 precursors and promote the release of IL-1β, IL18, and lactic acid, resulting in GSEMD cutting and forming holes in the cell membrane [14, 19]. Previous studies have reported that this form of cell death occurs in vascular lesions. Pyroptosis of vascular endothelial cells, macrophages, and smooth muscle cells can induce plaque instability and accelerate the progression of atherosclerosis. The upstream stimulus factors that induce pyroptosis are mainly involved in ROS production, mitochondrial damage, and autophagy [19], which have been demonstrated in our previous studies on smooth muscle cells in IAs [50, 51]. NLRP3 knockdown [52] and antagonism of inflammatory cytokines [30] have been effective in preventing the occurrence of abdominal aortic aneurysms in animal models. In particular, in terms of vascular subcellular localization, studies have revealed that AIM2, CASP1, and ASC [53] are located in smooth muscle cells [32] and macrophages. We observed a similar localization pattern during IHC staining of IA tissues, indicating that targeting the inflammasome and inducing pyroptosis of smooth muscle cells may be crucial factors promoting vascular wall degeneration and aneurysm formation. Although we did not include CASP5 in the validation study, CASP5, a member of the non-classical pyroptosis death pathway, also involving in the vascular inflammatory response. Unlike CASP1, little is known about the role of CASP5 in pyroptosis. Caspase-4/5/11 can be directly activated by LPS to complete GSEMD cleavage; however, this process still needs NLRP3-activated CASP1 [32]. The median numbers of AIM2 protein and mature caspase-5 (p20) were significantly increased in abdominal aortic samples associated with a high risk of rupture than in those associated with a low risk of rupture [53]. A recent study supported that caspase3, which is regarded as a marker for apoptosis, can transform apoptosis to pyroptosis by cutting GSDME [13]. Regulating the chemotaxis of immune cells to protect smooth muscle cells can be a therapeutic target to stabilize unruptured IAs. Therefore, further studies on the role of the above DEPRGs in cerebrovascular smooth muscle and immune cells will provide a deeper understanding of the mechanism underlying IA formation.

Adverse immune responses are closely associated with the disease course of IAs. To clarify the differences in immune cell types in UIAs, the CIBERSORT algorithm was used to analyze 22 types of immune cell infiltration in UIA tissues. The results indicated that infiltration of activated mast cells and CD8 T cells was significantly increased. Mast cells, traditionally called allergic response cells, are essential in cardiovascular diseases [54]. These cells, in response to external stimuli, function in combination with eosinophils to synthesize and release allergic substances [54, 55], including histamine, leukotrienes [56, 57], chymase, and tryptase [55, 58]; these substances induce inflammation and structural remodeling of blood vessel walls [55, 59]. Further, immune cell correlation analysis suggested that activated mast cells were positively correlated with eosinophils and CD8 T cells. The presence of mast cells is associated with aneurysm wall degeneration and microbleeding; this is consistent with our findings. Previous studies have reported an association between mast cell activation and pathological remodeling of aneurysm walls [4, 60]. Mast cell stabilizers such as cromolyn [4] and leukotriene inhibitors such as montelukast [61] reduce the incidence of aneurysms in animal models. In the present study, we also observed a significant differences in CD8 T cells. PD1, a key immune checkpoint receptor, is expressed on activated T cells [62]. PD1 and HLA-DR are highly upregulated in CD8 T cells, indicating T cell activation in IAs [63]. Furthermore, macrophages in our result contain a large proportion, which was also confirmed via single-cell sequencing of IAs in mice [61, 64]. Smooth muscle contractive dysfunction and phenotypic transformation are important pathological manifestations of intracranial aneurysms. Pyroptosis of macrophages releases immune factors and matrix metalloproteinases (MMP) [55], possibly leading to phenotypic changes in smooth muscles and pyroptosis and destruction of the ECM [65]. Extensive macrophage infiltration ultimately increases the risk of IA rupture [50, 66]. To our surprise, there were significantly higher M2 macrophages than M1 macrophages in IA tissues; this is beyond our expectation. In fact, during clinical follow-up, we noticed that not all IAs exhibited increased volume and ruptured hemorrhage. These aneurysms may not continuously progress and remain stable. Of course, hemodynamic differences among different patients and aneurysm location are also important aspects affecting the outcome of IA [67]. By detecting the changes in cytokine levels in the cerebrospinal fluid of patients with UIAs, Kamińska et al. concluded that proinflammatory and anti-inflammatory mechanisms may be simultaneously activated and that the expression of proinflammatory reactive proteins in the cerebrospinal fluid is positively correlated with the size and number of aneurysms [26]. This may partially explain the stabilizing effect of an increased number of M2 macrophages on unruptured aneurysms; however, the number of samples and individual differences may limit the accuracy of this result. In addition, we observed that although there was no significant difference between the UIAs and RIAs groups during validation studies using the GSE54083 [17] dataset, the expression of these pyrotposis-related hub genes was still higher in the RIAs group than in the UIAs and control groups. Therefore, we speculate that the cytokine storm may break the balance between proinflammatory and anti-inflammatory factors, triggering IA rupture. Overall, IAs may have complex immunomodulatory networks, and T cell, mast cells and macrophages may be important immune nodes that regulate the state of the IAs [68]. These immune cells may have a complex association with *NLRP3*, *IL1B*, and *IL18*. Their potential mechanisms in the immune system of IAs should be further verified in vivo and in vitro.

### Limitation

Our study has some limitations, such as small sample size of datasets and clinical tissues and large differences among samples, that should not be ignored. The subcellular localization of pyroptosis in IAs is still relativelyobscure. Moreover, we did not consider the type of IAs. Most of the specimens available in clinical settings are cystic aneurysms, whereas others include dissecting and blood blister-like aneurysms. There are differences in the formation of different types of IAs. Therefore, future studies should include a larger sample size, use animal models, and perform single-cell sequencing of human IAs to further confirm our results. Moreover, the association of pyroptosis-related hub genes with the risk of aneurysm rupture should be explored in prospective cohort studies.

## Conclusion

To sum up, we identified the pyroptosis-related hub genes in IAs by bioinformatics methods, and explored their inflammatory pathways and mechanisms. Further, the correlation between the expression profile of immune cells and these genes was revealed. Our study provides new insights into the molecular mechanism of pyroptosis-related IA formation and rupture.

### Supplementary Information

**Additional file 1. Table S1.** 51 Pyroptosis-related genes

**Additional file 2. Table S2.** Clinical information on tissue samples

**Additional file 3. Table S3.** The 13 differentially expressed pyroptosis-related genes in IA samples

**Additional file 4. Table S4.** The GSEA result

**Additional file 5. Table S5.** The results of GO and KEGG enrichment analysis

**Additional file 6. Table S6.** The Node table of PPI network. **Table S7. **The Edge table of PPI network. **Table S8.** The topological measurements of PPI network

**Additional file 7. Figure S1.** The soft threshold of WGCNA. **a** The scale-free fit index soft-thresholding powers. **b** The mean connectivity for various soft-thresholding powers. **c, d** Scatter plot of the cyan and the magenta modules.

**Additional file 8. Table S9.** Immunoinfiltration score. **Table S10**. Correlation analysis of 22 kinds of immune cells

## Data Availability

The GSE datasets that support the finding of this study are available in the GEO database (https://www.ncbi.nlm.nih.gov/geo/) with the following data accession identifiers: GSE76436 and GSE54083.

## Abbreviations

IA: Intracranial aneurysm
PRGs: Pyroptosis-related genes
DEPRGs: Differentially expressed PRGs
WGCNA: Weighted gene coexpression network analysis
IHC: Immunohistochemistry
AUC: Area under the curve
ECM: Extracellular matrix
GEO: Gene expression omnibus
GPL: GEO platform
FDR: False discovery rate
GSEA: Gene set enrichment analysis
KEGG: Kyoto Encyclopedia of genes and genomes
NES: The normalized enrichment score
PPI: Protein–protein interaction
MCODE: The molecular complex detection
EPC: Edge percolated component
MNC: Maximum neighborhood component
MCC: Maximal clique centrality
MMP: Matrix metalloproteinases
ROC: The receiver operating characteristic
UIAs: Unruptured intracranial aneurysms
RIAs: Ruptured aneurysms
