# Supplementary material for: Identifying pyroptosis- and inflammation-related genes in intracranial aneurysms based on bioinformatics analysis
Source: Biol Res. 2023 Sep 27;56:50. doi: 10.1186/s40659-023-00464-z (PMC10523789; doi:10.1186/s40659-023-00464-z)
Supplement: Supplementary file 1 — Additional file 1. Table S1. 51 Pyroptosis-related genes [file 40659_2023_464_MOESM1_ESM.docx]

Table S1 51 Pyroptosis-related genes^a^

| GSDMA | CASP4 | GBP3 | ZBP1 | NLRP2 | MEFV |
| --- | --- | --- | --- | --- | --- |
| GSDMB | CASP5 | GBP4 | PRF1 | NLRP3 | PYDC1 |
| GSDMC | CASP6 | GBP5 | DHX9 | NLRP6 | PYCARD |
| GSDMD | CASP8 | DDX3X | DFNB59 | NLRP7 | CARD16 |
| GSEME | GZMA | NAIP | CTSG | NLRP9 | CARD18 |
| IL1B | GZMB | SERPINB1 | APIP | NLRP12 | CARD8 |
| IL18 | HMGB1 | C-FLIP | AIM2 | NLRX1 |  |
| CASP1 | GBP1 | TNFRSF12 | NLRC4 | NOD2 |  |
| CASP3 | GBP2 | IRF2 | NLRP1 | TLR4 |  |

a Wang, N., et al., Molecular investigation of candidate genes for pyroptosis-induced inflammation in diabetic retinopathy. Front Endocrinol (Lausanne), 2022. 13: p. 918605.
