# Supplementary material for: Identifying pyroptosis- and inflammation-related genes in intracranial aneurysms based on bioinformatics analysis
Source: Biol Res. 2023 Sep 27;56:50. doi: 10.1186/s40659-023-00464-z (PMC10523789; doi:10.1186/s40659-023-00464-z)
Supplement: Supplementary file 2 — Additional file 2. Table S2. Clinical information on tissue samples [file 40659_2023_464_MOESM2_ESM.docx]

Table S2 Clinical information on tissue samples

|  | Gender | Age | Aneurysm type | Tissue source | Hypertension | Diabetes mellitus | Coronary heart disease |
| --- | --- | --- | --- | --- | --- | --- | --- |
| 1 | Female | 70 | UIA | R-MCA | Yes | Yes | Yes |
| 2 | Female | 55 | UIA | ACoA | Yes | No | No |
| 3 | Female | 55 | UIA | ACA | Yes | No | No |
| 4 | Male | 67 | UIA | R-MCA | Yes | No | No |
| 5 | Male | 56 | RIA | R-MCA | No | Yes | No |
| 6 | Female | 65 | RIA | L-PICA | Yes | No | No |
| 7 | Male | 48 | RIA | ACoA | Yes | No | No |
| 8 | Female | 74 | RIA | R-MCA | No | No | No |
| 9 | Male | 37 | STA | R-STA | No | No | No |
| 10 | Female | 56 | STA | L-STA | No | No | No |
| 11 | Male | 46 | STA | R-STA | Yes | Yes | No |
| 12 | Male | 49 | STA | L-STA | No | No | No |

UIA: Unruptured intracranial aneurysm; RIA: Ruptured intracranial aneurysm; STA: Superficial Temporal Artery.

ACoA: Anterior communicating artery; ACA: Anterior Cerebral Artery; PICA: Posterior Inferior Cerebellar Artery; MCA: Middle Ccerebral Artery; R: Right; L: Left.
