# Supplementary figures and images for: Identifying pyroptosis- and inflammation-related genes in intracranial aneurysms based on bioinformatics analysis
Source: Biol Res. 2023 Sep 27;56:50. doi: 10.1186/s40659-023-00464-z (PMC10523789; doi:10.1186/s40659-023-00464-z)

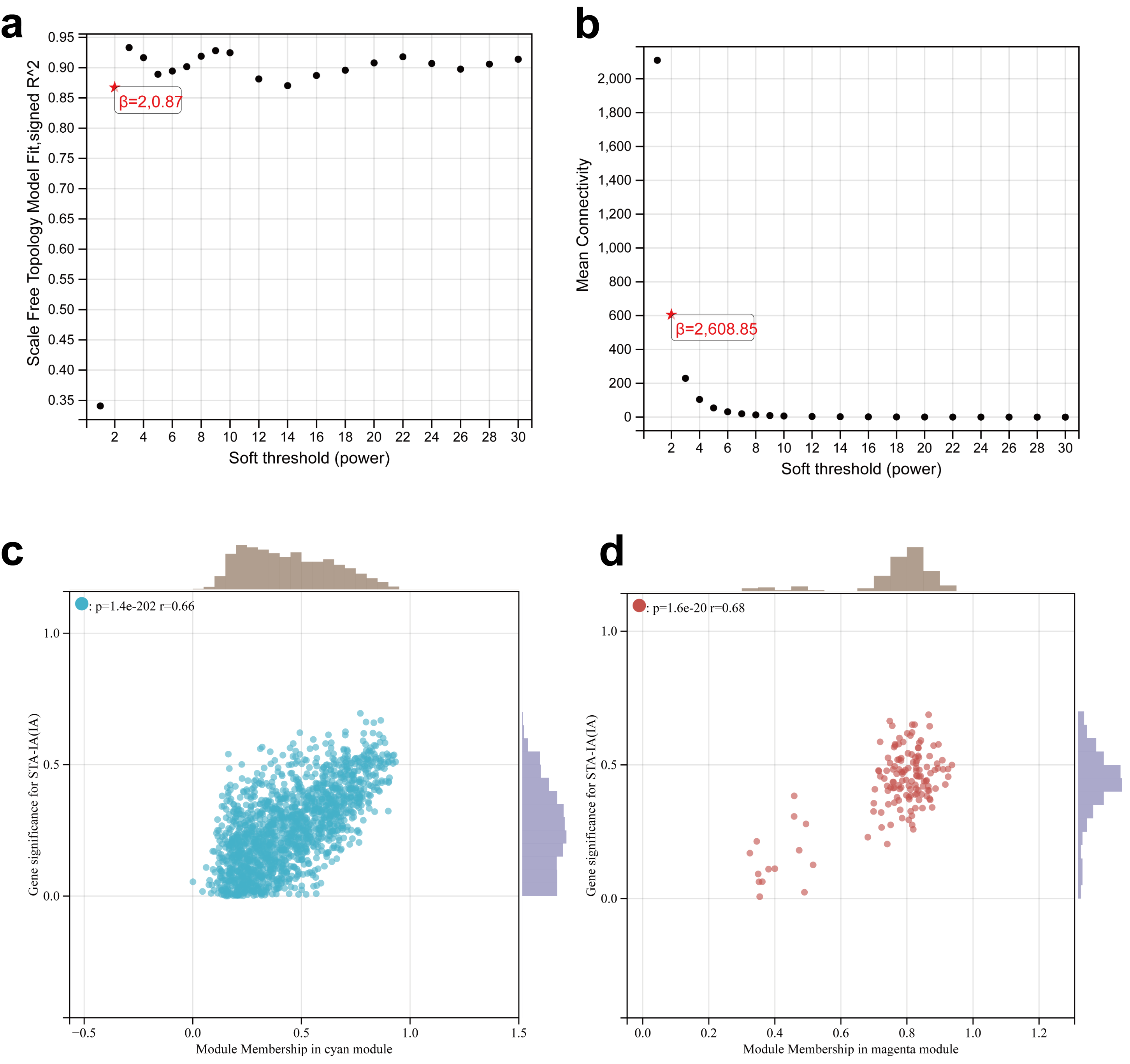

Supplement: Supplementary file 7 — Additional file 7. Figure S1. The soft threshold of WGCNA. a The scale-free fit index soft-thresholding powers. b The mean connectivity for various soft-thresholding powers. c, d Scatter plot of the cyan and the magenta modules. [file 40659_2023_464_MOESM7_ESM.tif]
